# Supplementary material for: Sculpting Windows onto AuAg Hollow Cubic Nanocrystals
Source: Nanomaterials (Basel). 2023 Sep 19;13(18):2590. doi: 10.3390/nano13182590 (PMC10538185; doi:10.3390/nano13182590)
Supplement: Supplementary file 1 [file nanomaterials-13-02590-s001.zip › nanomaterials-2588762-supplementary.pdf]

Supplementary information

Materials and methods.

Chemicals

Ethyleneglycol anhydrous 99.8% (EG), silver trifluoroacetate (CF_3COOAg), polyvinylpyrrolidone (PVP, MW: 55,000; 360,000), hydrochloric acid 37% (HCl), sodium hydrosulfide (NaHS), ascorbic acid (AA), gold (III) chloride trihydrate >99,9% ($HAuCl_4$. $3H_2O$), sodium citrate tribasic dihydrate (SC), cetyltrimethylammonium bromide (CTAB), cetyltrimethylammonium chloride (CTAC), cetyltrimethylammonium *p*-toluenesulfonate (CTApTS), bovine serum albumin (BSA), Anti-BSA (bovine serum albumin) antibody, were purchased from Sigma-Aldrich. All chemicals were used as received without further purification. Distilled water passed through a Millipore system (ρ = 18.2 mΩ) was used in all experiments.

Methods

Synthesis of Ag Nanocubes. Ag NCs were synthesized by a modified polyol method, briefly 15 mL of EG was added to a 100 mL round-bottomed flask, the flask was closed and then it was heated in a silicon oil bath at 150°C. The reaction solution was continuously stirred using a magnetic stir bar. After 10 min, 180 µL of 3mM NaHS solution in EG was added. After 2 min, 1.5 mL of 3 mM HCl solution in EG and 3.8 mL of 20 mg/mL PVP were added. Finally, after 3 min, 1.2 mL of 282 mM CF_3COOAg solution in EG was added. After 60 min, the reaction was stopped by placing the reaction flask in an ice-water bath. Resultant Ag NCs were purified by centrifugation (8000 g, 20 min) in order to remove the EG and the excess of PVP, and further dispersed in MQW before sample characterization.

Synthesis of bimetallic AgAu hollow nanostructures. Synthesis of AgAu hollow nanostructures. Bimetallic hollow AgAu nanostructures were synthesized via GRR and/or Kirkendall effect at room temperature. In a typical procedure, 0.25 mL of Ag NCs (~10^{12} NPs/mL) were dispersed in 1 mL of MQW, 1 mL of surfactant (CTAB, CTAC, CTApts 14 mM or PVP), and 0.1 mL of 1 mM AA, were added. Then, increasing amounts of $HAuCl_4$ (1 mM), was added through a syringe pump at a rate of 25 µL/min under constant stirring. After the addition of the $HAuCl_4$ solution, the reaction was stirred for about 30 min at room temperature until the UV–vis spectra of the solution became stable. The sample was centrifuged at 8000 g for 10 min and the supernatant was discarded. The pellet was suspended in 1 mL of MQW for further characterization.

Synthesis of AgAu hollow nanostructures with SPR band at 1000nm. In a typical procedure, 250 µL of Ag NCs (~10^{12} NPs/mL) were dispersed in 2 mL of PVP 20 mg/mL (180 mM by repeating unit) and 0.1 mL of AA 0.1 mM were added. Then, 250 µL of $HAuCl_4$ (1 mM), was added through a syringe pump at a rate of 10 µL/min under constant stirring. After the addition of the $HAuCl_4$ solution the reaction was stirred for 30 min at room temperature until the UV–vis spectra of the solution became stable. The sample was then centrifuged at 8000 g for 10 min and the supernatant was discarded. The pellet was suspended in 1 mL of MQW for further characterization.

Characterization. Absorption spectra of the as-synthesized NCs were acquired with a Shimadzu UV-2401 PC or a Perkin Elmer Lambda 25 spectrophotometer. An aliquot of the NCs solution was placed in a cuvette, and spectral analysis was performed at room temperature. The morphology and size of the NCs were visualized using FEI Magellan 400L XHR SEM, in transmission mode operated at 20 kV. TEM, HR-TEM, and STEM-HAADF images were obtained from a FEI Tecnai G2 F20 S-TWIN HR(S) TEM, operated at an accelerated voltage of 200 kV. A droplet of the sample was drop cast onto a piece of ultrathin carbon-coated 200-mesh copper grid (Ted-pella, Inc.) and left to dry in air. XRD data were collected on a PANalytical X'Pert diffractometer using a Cu Kα radiation source.

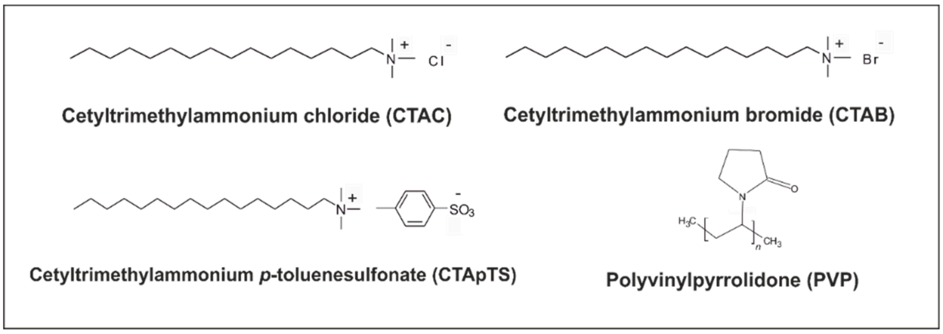

Figure S1. Structure of surfactant used in this study.

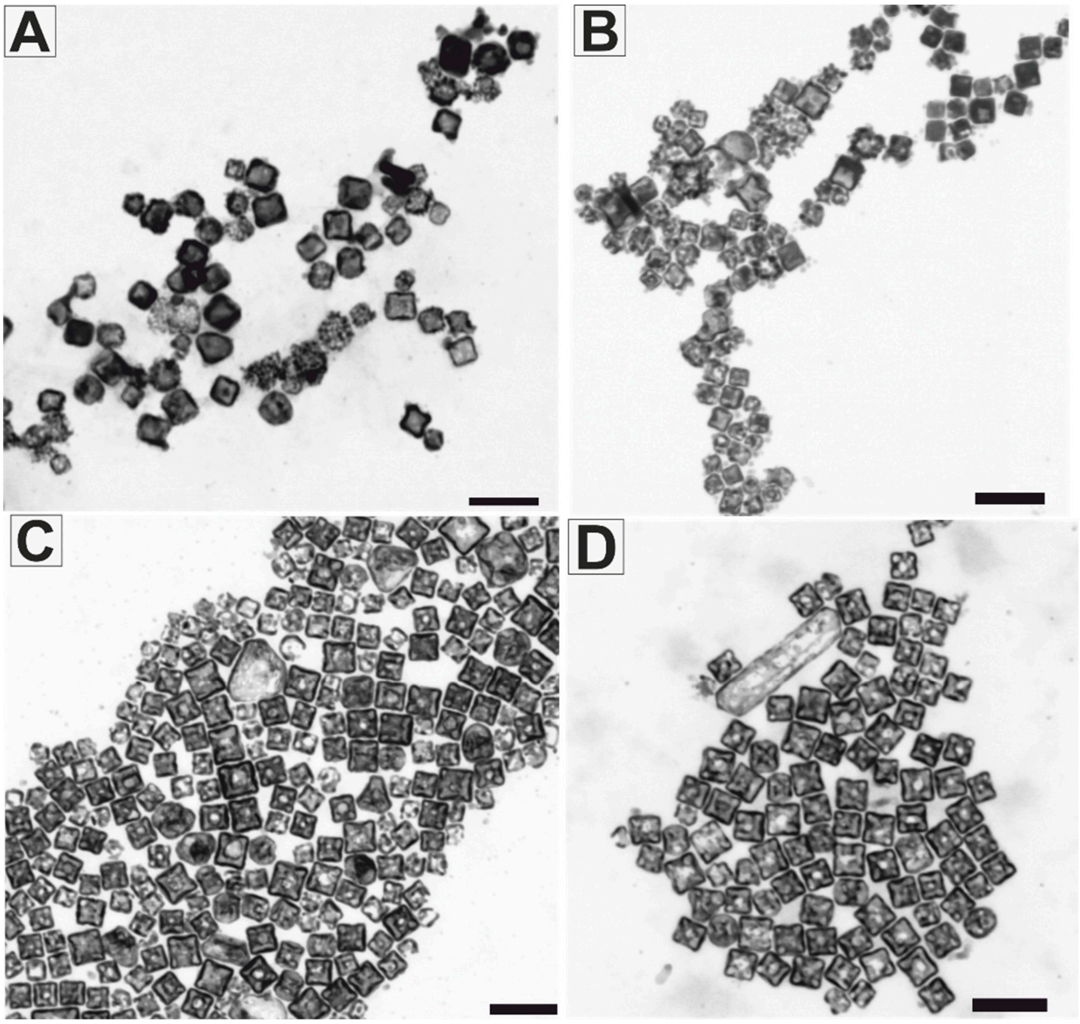

Figure S2. Effect of CTAB concentration**. A.** 0.14 mM **B.** 1.4 mM **C.** 14 mM and **D.** 140 mM. Scale bar 100 nm for all images.

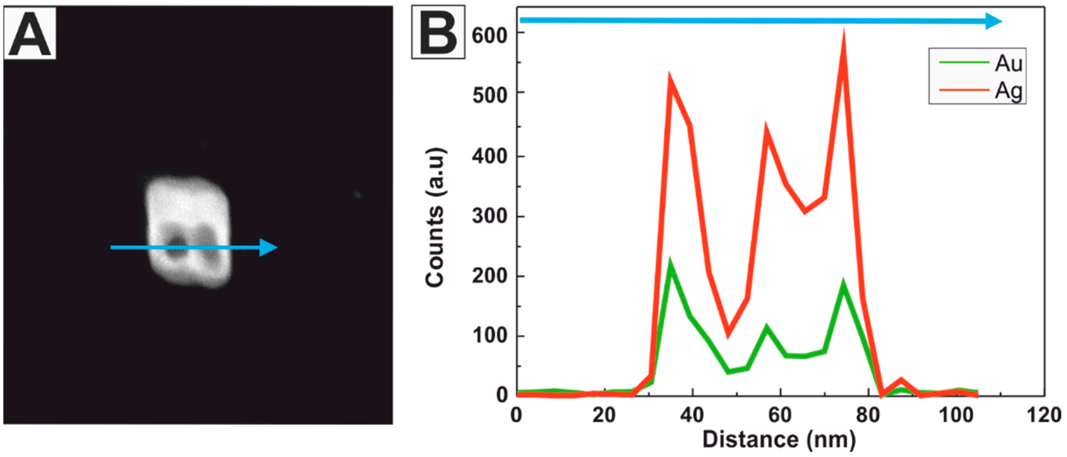

Figure S3 (A). HAADF-STEM image of a two pinholed NC. **B.** EDS line scanning through the blue arrow of the double pinholed NC presented in **A.**

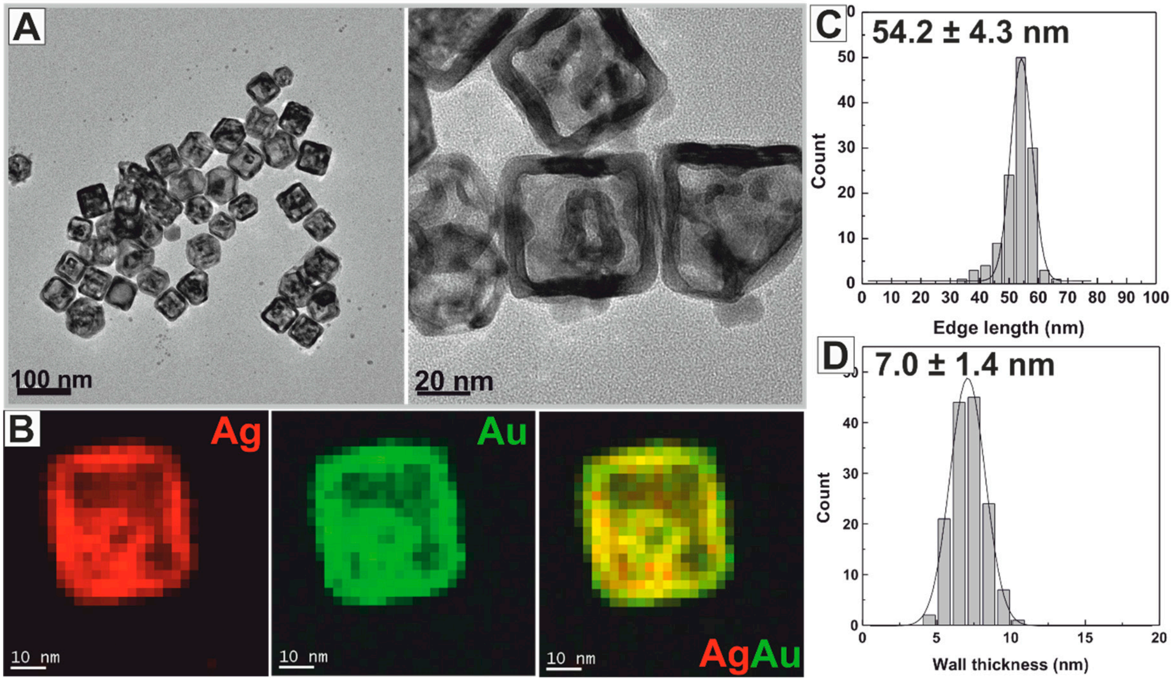

Figure S4. A.TEM images of double walled nanoboxes obtained when CTAC was used as surfactant. **B.** EDS mapping of a double walled nanobox, red for Ag, green for Au and composite. **C.** Size distribution and **D.** Wall thickness distribution

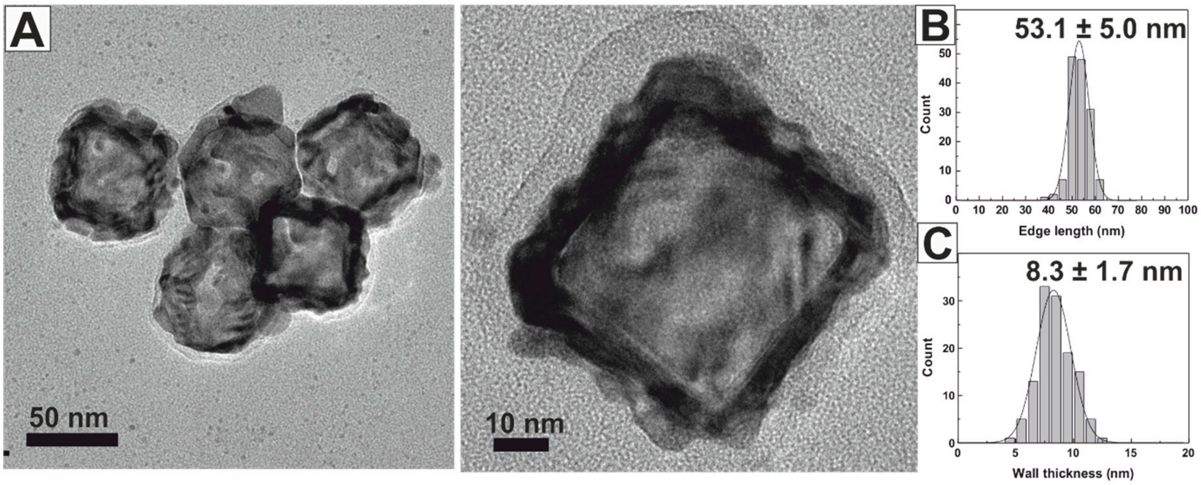

Figure S5. TEM images of single walled nanoboxes synthesized in the presence of CTApTS. **B.** Size distribution and **C.** Wall thickness of single walled nanoboxes.
